# Protective effects of triple fermented barley extract (FBe) on indomethacin-induced gastric mucosal damage in rats

**DOI:** 10.1186/s12906-019-2457-0

**Published:** 2019-02-20

**Authors:** Jong-Min Lim, Chang-Hyun Song, Su-Jin Park, Dong-Chan Park, Go-Woon Jung, Hyung-Rae Cho, Khawaja Muhammad Imran Bashir, Sae Kwang Ku, Jae-Suk Choi

**Affiliations:** 1 Glucan Corp., #305 Marine Bio-Industry Development Center, Hoenggye-ri 27, Ilgwang-myeon, Gijan-gun, Busan, 46048 Republic of Korea; 2 Department of Anatomy and Histology, College of Korean Medicine, Daegu Haany University, 290 Yugok-dong, Gyeongsan-si, Gyeongsanbuk-do 38610 Republic of Korea; 3 MRC-GHF, College of Korean Medicine, Daegu Haany University, 290 Yugok-dong, Gyeongsan-si, Gyeongsanbuk-do 38610 Republic of Korea; 4 Research Center for Extremophiles and Microbiology, College of Medical and Life Sciences, Silla University, 140, Baegyang-daero 700 beon-gil, Sasang-gu, Busan, 46958 Republic of Korea; 5 Research Center for Life Science Technologies in Medicine and Environment, 31, Gwahaksandan 1-ro, 60 beon-gil, Gangseo-gu, Busan, 46742 Republic of Korea; 6 Division of Bioindustry, College of Medical and Life Sciences, Silla University, 140, Baegyang-daero, 700 beon-gil, Sasang-gu, Busan, 46958 Republic of Korea

**Keywords:** Barley extract, Fermentation, Gastric ulcer, Gastroprotection, *Hordeum vulgare*, Indomethacin

## Abstract

**Background:**

*Hordeum vulgare* L (barley) contains numerous phenolic substances with proven anticancer, antioxidant and gastroprotective activities. Saccharification increases the functionality and bioavailability of these compounds thus can aid in the development of a natural product based medicine. This study aimed to investigate the possible gastroprotective effects of saccharification on the indomethacin (IND)-induced gastric ulcers in rats using *Weissella cibaria*- and *Saccharomyces cerevisiae*-triple fermented *H. vulgare* extract (FBe).

**Methods:**

In total, 60 healthy male 6-week old Sprague-Dawley SD (SPF/VAF Outbred CrljOri:CD1) rats were commercially purchased. The FBe extract (100, 200, and 300 mg kg^− 1^) was orally administered 30 min before an oral treatment of IND (25 mg kg^− 1^). Six hours after IND treatment, variations in the histopathology, myeloperoxidase (MPO) activity, gross lesion scores, lipid peroxidation, and antioxidant defense system component (superoxide dismutase (SOD), catalase (CAT), and glutathione (GSH)) levels were measured.

**Results:**

FBe treatment showed significant (*p* < 0.01 or *p* < 0.05) and dose-dependent decrease in gastric mucosal damage. In the present study hemorrhagic gross lesions, gastric MPO activity, and histopathological gastric ulcerative lesions were observed in IND-treated rats compared to the IND control rats. In particular, FBe, in a dose-dependent manner, strengthened the antioxidant defense systems, decreased lipid peroxidation and CAT activity by increasing the GSH levels and SOD activity, respectively. The 200 mg kg^− 1^ dose of FBe was similarly gastroprotective as the 10 mg kg^− 1^ dose of omeprazole in rats with IND-induced gastric mucosal damage.

**Conclusions:**

The findings of the present study show that an oral administration of FBe had positive gastroprotective effects through strengthening the body antioxidant defense system and anti-inflammatory effects.

## Background

Occurrence of gastric ulcer has been associated with numerous factors such as imbalance between protective and aggressive factors, pulmonary and liver diseases, alcohol consumption, and non-steroidal and steroidal drugs [1, 2]. Due to favorable preventive effects in instances of malignant tumors, eclampsia, dementia, and hyperlipidemia, the demand for non-steroidal anti-inflammatory drugs (NSAIDs) has remarkably increased in recent years [3]. However, NSAIDs have also been linked with risks of adverse gastrointestinal disorders including gastric mucosal ulceration, erosion, bleeding, and perforation [4]. About 25% of urgent gastric ulcers have been related to NSAIDs administration [5], with various factors like stress and *Helicobacter pylori* infections exacerbating the condition related to gastric ulcers [6].

Various synthetic anti-ulcer drugs such as misoprostol are used to cure NSAID induced gastric ulcers. Similarly, indomethacin (IND) is widely approved in medical practice as being an NSAID; it shows exceptional efficiency in the treatment of fever, pain and inflammation by suppressing the synthesis of prostaglandins through inhibiting the cyclooxygenase enzymes [7]. However, NSAIDs such as IND administration result in gastrointestinal tract infections due to the inhibition of prostaglandin synthesis [8]. In addition, IND generates harmful reactive oxygen species (ROS) involved in pathogenesis of gastric ulcers [9]. Apparently, the free radical scavenging property of synthetic drugs might have a protecting effect against gastric mucosal oxidative damage that accelerates healing of gastric ulcers [10]. However, disorder of gastric mucosal antioxidant defense system has also been associated with NSAIDs [11].

Investigating dietary plants that are valued in traditional medicine might hold promise for prolonged use. Functional foods originating from natural sources are gaining significance in the pharmaceutical industry. Fermented herb extracts have been widely used as a source of bioactive compounds in pharmaceutical and food industries as bioactivity of natural herbs increases during fermentation through biotransformation or probiotic effect [12–14]. Fermented barley extracts have revealed effective pharmacological effects including antioxidant [15], anti-atopic dermatitis [16], uric acid lowering [17], hepatoprotective [15], and immunostimulatory [18] activities. Potent anti-ulcer agents from natural herbs with strong antioxidant effects, such as *Artemisia asiatica* extract (Stillen™, Dong-A Pharmaceuticals, Yongin, Rep. of Korea) have also appeared in the market [19].

It is noted that fermented barley extract shows potent antioxidant potential in various in vivo models [20, 21], and indomethacin induces gastric ulcer in Sprague-Dawley animal models [6, 10]. This gained our attention to address the possible protective effect of fermented barley against IND-induced gastric ulcers in Sprague-Dawley rat model. Previously, we reported triple fermentation of barley using saccharification with *Weissella* and *Saccharomyces* as an effective and valuable fermentation choice [22–24] with less toxicity [25], and the present research intended to estimate the healing effect of triple fermented barley extract (FBe) on the IND-induced gastric ulcers in Sprague-Dawley rats, a representative valuable animal model to screen for gastroprotective agents [6, 10].

## Methods

### Experimental animals and husbandry

**Fig. 1:**
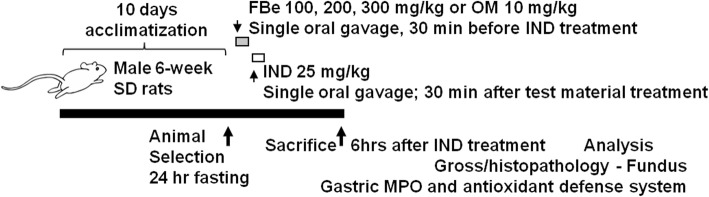
Experimental designs used in this study. FBe: triple fermented barley extract as test material; IND: indomethacin; OM: omeprazole

Sixty healthy male 6-week old Sprague-Dawley SD (SPF/VAF Outbred CrljOri:CD1) rats purchased from OrientBio Co., Seungnam, Republic of Korea, were used in this study following acclimatization for 10 days. Animals were raised in polycarbonate cages at 20–25 °C and a relative humidity of 30–35%. The rats were subjected to 12-h photoperiod while food and water was freely accessible. With six groups and eight rats in each group, 48 rats were selected based on the body weight (average 258.58 ± 15.62 g, ranged in 229.00–283.00 g) measured one day before test material administration and used for the experiments. Experimental groups (eight rats in each of the six groups) are presented in Fig. 1.

The animal experiments were conducted according to the international regulations of the usage and welfare of laboratory animals and were approved by the Institutional Animal Care and Use Committee, Daegu Haany University, Gyeongsan, Rep. of Korea [Approval No: DHU2014–087].

### Compositions of the fermented barley extract (FBe)

Nutritional facts, including calories, carbohydrates, proteins, lipids, and dietary fiber levels were evaluated, according to the method of Food Code [26]. Total polyphenols and total flavonoids were evaluated according to the method of Health Functional Food Code [27].

### Preparations and administration of test materials

The triple fermented barley extract (FBe) was provided by Glucan Corporation (Busan, Rep. of Korea). The FBe extract was prepared in three different steps. In saccharification (the first step), 1 kg non-glutinous barley was washed, soaked (6 h), drained (30 min), and steamed (15 min at 121 °C). After cooling, malt solution [10 g malt powder in 4 L distilled water] was added to the mixture and fermented for 12 h at 55 °C in a 20 L glass bioreactor. In the second step, 20 mL suspension of *Saccharomyces cerevisiae* (ATCC, VA, USA) was added to the first fermentate, mixed thoroughly, and fermented again at 30 °C for 48 h. In the last step, 20 mL of *Weissella cibaria* (lactic acid bacteria; KACC, Suwon, Rep. of Korea) was added to the second fermentate, mixed thoroughly, and fermented third time at 30 °C for 48 h. The prepared third fermentate was sterilized (150 °C for 15 min) and sieved through a 40-mesh sieve to get the final product (triple fermented barley extract; FBe). Some specimens of FBe [Code: FBe2014Ku01] were deposited in the herbarium of the Medical Research Center for Globalization of Herbal Formulation, Daegu Haany University, Gyeongsan, Rep. of Korea. The final product was stored at 4 °C to protect from moisture and light. FBe solutions (100, 200, and 300 mg kg^− 1^) were prepared by dissolving in distilled water (DW) and orally administered once (in a volume of 5 mL kg^− 1^ of rat) 30 min before IND (25 mg kg^− 1^ in a volume of 5 mL kg^− 1^ of rat) administration. Omeprazole (10 mg kg^− 1^; OM; Sigma-Aldrich, St. Louis, MO, USA) was used as a reference drug [26]. To provide the similar experimental settings and administration stress, IND and intact control rats were once orally administered with 5 mL kg^− 1^ (*v*/*w*) of DW instead of the test material (Fig. 1).

### Induction of gastric mucosal damages by IND in rats

Thirty minutes after administration of vehicle, dosages of FBe or OM on 24 h fasted rats, IND (Sigma-Aldrich) was single orally administered at a dosage of 25 mg kg^− 1^ of rat [28]. In intact controls, only sterilized DW (5 mL kg^− 1^) was administered once by gastric gavages instead of IND (Fig. 1).

### Quantification of gross lesions

The rats were sacrificed by cervical dislocation under anesthesia using 99.50% CO_2_ gas 6 h after treatment with IND or vehicle. The stomach was excised after a median incision, fixed in 10% neutral buffered formalin for 24 h, and acquired digital images. The ulcer areas on the stomach surface were estimated by an automated image analyzer (IMT i-solution Inc., Quebec, Canada) as reported by Suleyman et al. [28]. Gastric damage scores were calculated macroscopically by measuring the visible lesions.

### Determination of gastric myeloperoxidase contents

A 0.2 g of the tissue sample was homogenized in 10 volumes of ice-cold potassium phosphate buffer [50 mM K_2_HPO_4_, pH 6.0 + 0.5% hexadecyltrimethylammonium bromide (HETAB)], and centrifuged at 12,000×g for 10 min at 4 °C [29]. Supernatant was discarded and the pellet was re-suspended in equal volumes of potassium phosphate buffer and 10 mM EDTA. One unit of myeloperoxidase (MPO) activity was defined as the amount of MPO g^− 1^ of tissue that changed an absorbance of 1.0 per min at 460 nm and 37 °C [30].

### Determination of malondialdehyde (MDA) formation

Thiobarbituric acid method was used to determine the concentration of gastric mucosal lipid peroxidation [31]. The corpus mucosa of stomach was scraped and homogenized in 10 mL of KCl solution (100 g L^− 1^). Five hundred microliter of the homogenate was added into a new tube containing thiobarbituric acid solution [1.5 mL of 8 g L^− 1^ 2-thiobarbiturate (Sigma-Aldrich), 0.2 mL of 80 g L^− 1^ SDS, 1.5 mL of 200 g L^− 1^ acetic acid, and 0.3 mL of DW], heated at 98 °C for 1 h, cooled at room temperature and added 5 mL of n-butanol: pyridine (15:1; Sigma-Aldrich). The mixture was vortexed, centrifuged at 3000×g for 30 min, and measured the absorbance of the supernatant by UV/Vis spectrophotometer at 532 nm. The 1,1,3,3-tetramethoxypropane (Sigma-Aldrich) was used to draw the standard curve and the results were displayed as nM of MDA g^− 1^ of wet tissue (nM/g tissue).

### Estimation of gastric mucosal glutathione (GSH) content

The GSH content was estimated by following a previously reported method of Sedlak and Lindsay [32]. The stomach’s mucosal surface was homogenized in 2 mL of Tris-HCl buffer [50 mM Tris HCl, 0.2 mM sucrose, and 20 mM EDTA; pH 7.5], precipitated with 0.1 mL of 25% trichloroacetic acid and centrifuged at 3500×g for 40 min at 4 °C to remove the precipitate. The GSH content was determined in the supernatant using 2-nitrobenzoic acid (Sigma-Aldrich) by measuring the absorbance at 412 nm and the results were expressed as nM/mg tissue.

### Tissue catalase (CAT) activity

The CAT activity of tissue was calculated by following a previously reported study of Evans and Diplock [33]. Rat gastric mucosal homogenate was diluted with buffer as described before. The CAT dilution (2 mL) was mixed with 1 mL of 30 mM H_2_O_2_ and the absorbance was measured at 240 nm. The CAT activity was expressed in mM/min/mg of tissue.

### Tissue superoxide dismutase (SOD) activity

The SOD activity was examined by following the previously reported methods of Bashir et al. [34] with slight modifications. Briefly, gastric homogenate (15 μl) was mixed with ethanol (250 μl), chloroform (125 μl), and cold deionized water (450 μl). The homogenate mixture was then centrifuged at 8000×g for 2 min at 4 °C and the extract (500 μl) was added to the reaction mixture [16% Triton X-100 (100 μl), 0.9 mM nitroblue tetrazolium (250 μl), and 500 μl of 72.4 mM triscacodylate buffer with 3.5 mM diethylene pentaacetic acid (pH 8.2)], incubated for 5 min at 37 °C, added 10 μl of 9 mM pyrogallol and incubated again for 5 min at 37 °C. After stopping the reaction with 2 M formic buffer, absorbance was measured at 540 nm. The SOD activity was expressed in mM/min/mg of tissue.

### Histopathology

Regions of individual stomach samples, the fundus between the cardiac and pylorus were crossly trimmed based on the lumen, fixed in 10% neutral buffered formalin for 24 h, and paraffin-embedded sections (3–4 μm) were prepared. Selected sections were examined under a light microscope after staining with hematoxylin and eosin. The histological profiles of changes in total thicknesses of fundic mucosa and individual cross trimmed fundus tissues were observed under a microscope at a resolution of 400 ×. Furthermore, fundus lesion invasive percentage was also calculated according to the eq. (1) and semiquantitative scoring was assigned as follows; 0 = normal intact mucosa, 1 = slight surface erosive damages, 2 = moderate mucosa damages and 3 = severe total mucosa damages, based on general and histomorphometric analysis [35]. The histopathologist was blinded to the group distribution during analyses.

1 $$ \mathrm{Invasive}\ \mathrm{percentage}\ \mathrm{of}\ \mathrm{lesions}\ \left(\%\right)=\left(\frac{\mathrm{Length}\ \mathrm{of}\ \mathrm{lesions}\ \mathrm{on}\ \mathrm{the}\ \mathrm{crossly}\ \mathrm{trimmed}\ \mathrm{fundic}\ \mathrm{walls}}{\mathrm{Total}\ \mathrm{thickness}\ \mathrm{of}\ \mathrm{crossly}\ \mathrm{trimmed}\ \mathrm{fundic}\ \mathrm{walls}}\right)\ \mathrm{x}\ 100 $$

### Statistical analyses

The experimental data were expressed as mean ± standard deviation (S.D.) of 8 rats. The variance homogeneity was estimated by the Levene’s test [36]. In case of insignificant deviation, the significance between different pairs was analyzed by one-way ANOVA using LSD. In the case of significant deviation, a non-parametric comparison test (Kruskal-Wallis H test) was used to compare the groups. If a significant difference was observed by the Kruskal-Wallis H test, the significance between specific pairs was estimated by the Mann-Whitney U test. Statistical analyses were performed using SPSS ver. 14 (IBM-SPSS Inc., Chicago, IL, USA) and the results were considered statistically significant at *p* < 0.05 and *p* < 0.01. Furthermore, to understand the efficacy of the tested substances, the percent changes between IND, FBe and OM treated rats were calculated by Eqs. (2) and (3), respectively [37].

2 $$ \mathrm{Changes}\ \mathrm{as}\ \mathrm{compared}\ \mathrm{with}\ \mathrm{the}\ \mathrm{intact}\ \mathrm{control}\ \left(\%\right)=\left\{\frac{\left(\mathrm{Data}\ \mathrm{of}\ \mathrm{IND}\ \mathrm{control}\ \mathrm{rats}-\mathrm{Data}\ \mathrm{of}\ \mathrm{intact}\ \mathrm{control}\ \mathrm{rats}\right)}{\mathrm{Data}\ \mathrm{of}\ \mathrm{intact}\ \mathrm{control}\ \mathrm{rats}}\right\}\ \mathrm{x}\ 100 $$

3 $$ \mathrm{Changes}\ \mathrm{as}\ \mathrm{compared}\ \mathrm{with}\ \mathrm{the}\ \mathrm{IND}\ \mathrm{control}\ \left(\%\right)=\left\{\frac{\left(\mathrm{Data}\ \mathrm{of}\ \mathrm{test}\ \mathrm{substance}\ \mathrm{treated}\ \mathrm{rats}-\mathrm{Data}\ \mathrm{of}\ \mathrm{IND}\ \mathrm{control}\ \mathrm{rats}\right)}{\mathrm{Data}\ \mathrm{of}\ \mathrm{IND}\ \mathrm{control}\ \mathrm{rats}}\right\}\ \mathrm{x}\ 100 $$

## Results

### Compositions of the fermented barley extract (FBe)

Nutritional facts including calories, carbohydrates, proteins, lipids, and dietary fiber were 385.3 kcal 100 g^− 1^, 93.0, 3.1, 0.1, and 20.20%, respectively. Total polyphenols and total flavonoids contents were 3.66 mg g^− 1^ and 0.31 mg g^− 1^, respectively (Table 1).

**Table 1:** Composition of fermented barley extract (FBE)

Nutrition fact	Unit	Amount
Calorie	kcal/100 g	385.3
Carbohydrates	%	93.0
Protein	%	3.1
Lipids	%	0.1
Total polyphenols	mg/g	3.66 ± 0.12
Total flavonoids	mg/g	0.31 ± 0.02
Dietary fiber	%	20.20

### Changes in the gastric mucosal lesions

**Fig. 2:**
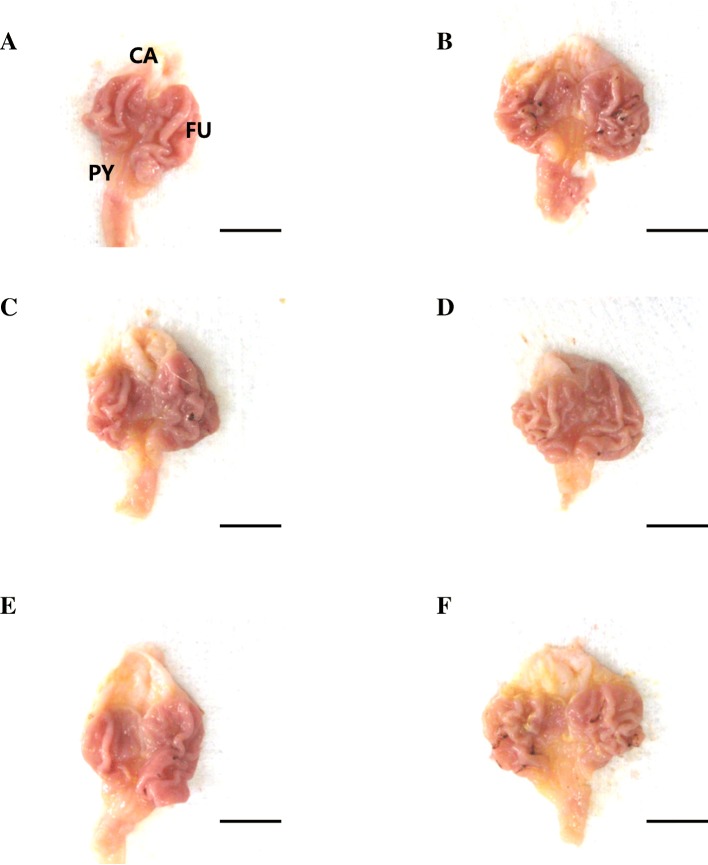
Representative gross stomach images taken from intact or IND-treated mice. **a**: Intact control (DW 5 ml kg^− 1^, twice administered rats with 30 min-intervals). **b**: IND control (DW 5 ml kg^− 1^ and IND 25 mg kg^− 1^ administered gastric mucosal damage induced vehicle control rats). **c**: OM (OM 10 mg kg^− 1^ and IND 25 mg kg^− 1^ administered reference drug treated rats). **d**: FBe 300 (FBe 300 mg kg^− 1^ and IND 25 mg kg^− 1^ administered the highest dosage experimental rats). **e**: FBe 200 (FBe 200 mg kg^− 1^ and IND 25 mg kg^− 1^ administered the middle dosage experimental rats). **f**: FBe 100 (FBe 100 mg kg^− 1^ and IND 25 mg kg^− 1^ administered the lowest dosage experimental rats). FBe: triple fermented barley extract as test material; DW: Distilled water; IND: indomethacin; OM: omeprazole; CA: cardiac regions of stomach; FU: fundus regions of stomach; PY: pylorus regions of stomach. All test substances were single orally administered, at 30 min before IND 25 mg kg^− 1^ single oral treatment, and all animals were sacrificed at 6 h after IND treatment. Scale bars = 11 mm

**Fig. 3:**
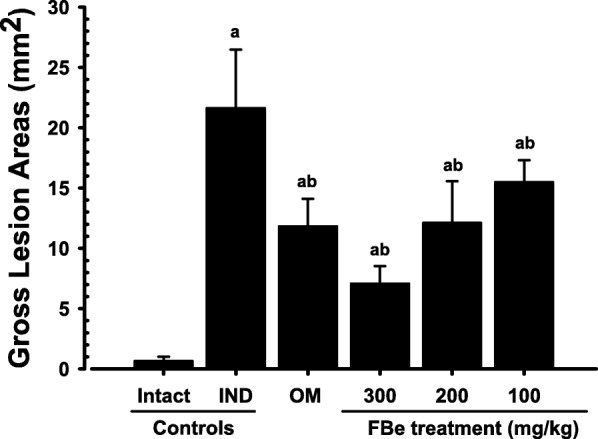
Gross lesion areas on the gastric mucosa, taken from intact or IND-treated rats. Values are expressed as mean ± S.D. of eight rats, mm^2^. FBe: triple fermented barley extract as test material; IND: indomethacin; OM: omeprazole. All test substances were single orally administered, at 30 min before IND 25 mg kg^− 1^ single oral treatment, and all animals were sacrificed at 6 h after IND treatment. ^a^*p* < 0.01 as compared with intact control by MW test. ^b^*p* < 0.01 as compared with IND control by MW test

Slightly insignificant restricted ulcerative lesions were observed in intact control rats while focal hemorrhagic ulcerative lesions in IND treated rats were grossly dispersed in the gastric mucosa. Marked inhibitions of the gross gastric damages were noticed in OM (10 mg kg^− 1^) and FBe (100, 200, and 300 mg kg^− 1^) treated rats. Accordingly, a significant increase in gastric mucosal gross lesion areas was noticed in IND control groups compared with intact control groups, however, mucosal lesions significantly decreased in a dose-dependent manner when treated with FBe (100, 200, and 300 mg kg^− 1^) or OM (10 mg kg^− 1^). An inhibitory effect on the increase in IND-induced gastric mucosal gross lesion areas was noticed in groups treated with FBe (200 mg kg^− 1^) or OM (10 mg kg^− 1^; Figs. 2 and 3).

### Effects on the gastric MPO content

**Fig. 4:**
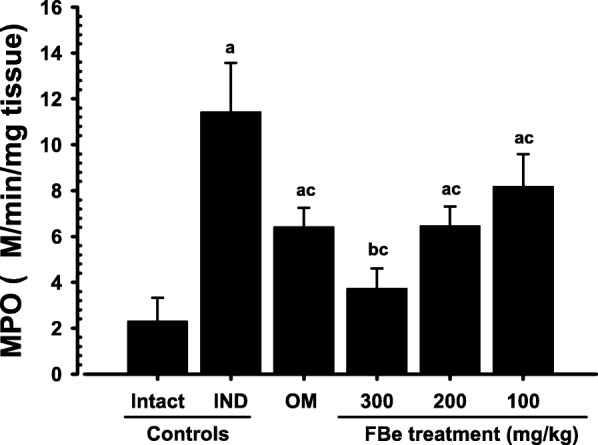
Gastric MPO contents in the intact or IND-treated rats. Values are expressed as mean ± S.D. of eight rats, μM/min/mg tissue. FBe: triple fermented barley extract as test material; IND: indomethacin; OM: omeprazole; MPO: myeloperoxidase. All test substances were single orally administered, at 30 min before IND 25 mg kg^− 1^ single oral treatment, and all animals were sacrificed at 6 h after IND treatment. ^a^*p* < 0.01 and ^b^*p* < 0.05 as compared with intact control by MW test. ^c^*p* < 0.01 as compared with IND control by MW test

IND control groups showed a noteworthy increase in gastric MPO content compared to the intact control groups. A significant and dose-dependent decrease in MPO content was observed in rats treated with FBe (100, 200, and 300 mg kg^− 1^). Furthermore, increase in IND-induced gastric MPO content was suppressed after a single oral application of OM (10 mg kg^− 1^) which was similar to the inhibition observed by treatment with FBe (200 mg kg^− 1^; Fig. 4).

### Effects on the lipid peroxidation

IND control rats showed increases in gastric lipid peroxidation compared to the intact vehicle control rats. Whereas, significant and dose-dependent decreases in lipid peroxidation were observed in FBe (100, 200, and 300 mg kg^− 1^) administered rats. Furthermore, IND-induced gastric lipid peroxidation was significantly suppressed after a single oral application of OM (10 mg kg^− 1^) which was similar to the inhibition observed by treatment with FBe (200 mg kg^− 1^; Table 2).

**Table 2:** Gastric lipid peroxidation, glutathione contents, CAT, and SOD activities in the intact or IND-treated rats

Items (Unit) groups	Lipid peroxidation (nM of MDA/g tissue)	Glutathione contents (nM/mg tissue)	Enzyme activity	
			CAT (mM/min /g tissue)	SOD (mM/min/mg tissue)
Control				
Intact	2.73 ± 1.15	5.21 ± 1.34	87.75 ± 17.29	151.50 ± 16.58
IND	20.02 ± 3.60^a^	1.49 ± 0.22^d^	174.25 ± 26.86^a^	75.75 ± 12.63^a^
Reference				
OM 10 mg kg^− 1^	12.91 ± 2.96^ac^	2.37 ± 0.42^d,e^	126.63 ± 14.92^a,c^	113.88 ± 10.74^a,c^
FBe treated as				
100 mg kg^− 1^	15.56 ± 1.95^a,c^	2.05 ± 0.34^d,e^	142.13 ± 13.42^a,c^	100.38 ± 18.91^a,c^
200 mg kg^− 1^	13.01 ± 2.16^a,c^	2.56 ± 0.68^d,e^	124.75 ± 19.31^a,c^	115.00 ± 11.99^a,c^
300 mg kg^− 1^	6.11 ± 1.79^a,c^	3.37 ± 0.54^d,e^	109.63 ± 16.40^b,c^	131.00 ± 15.15^a,c^

Values are expressed as mean ± S.D. of eight rats

*FBe* triple fermented barley extract as test material, *IND* indomethacin, *OM* omeprazole, *MDA* malondialdehyde, *CAT* catalase, *SOD* superoxide dismutase

^a^*p* < 0.01

^b^*p* < 0.05 as compared with intact control by LSD test

^c^*p* < 0.01 as compared with IND control by LSD test

^d^*p* < 0.01 as compared with intact control by MW test

^e^*p* < 0.01 as compared with IND control by MW test

### Changes in the GSH contents

Statistically significant decreases in endogenous antioxidants and gastric GSH levels were noticed in IND control rats compared to the intact control rats, but these decreases in GSH levels induced by treatment with IND were significantly suppressed after a single oral application of FBe (100, 200, and 300 mg kg^− 1^) or OM (10 mg kg^− 1^). FBe (200 mg kg^− 1^) revealed inhibitory effect on the decrease in IND-induced gastric GSH levels which was similar to those observed by treatment with OM (10 mg kg^− 1^; Table 2).

### Changes on the CAT activities

IND control rats showed significant (*p* < 0.01) increases in gastric CAT activity as compared to the intact control rats, but these elevations of CAT activities induced by treatment of IND were significantly suppressed after a single oral application of FBe (100, 200, and 300 mg kg^− 1^) or OM (10 mg kg^− 1^). FBe at a dosage of 200 mg kg^− 1^revealed similar inhibitory effect on the increase in IND-induced gastric CAT activities compared to those observed by treatment with OM (10 mg kg^− 1^; Table 2).

### Effects on the SOD activities

IND control groups showed significant decreases in gastric SOD activities compared to the intact vehicle control groups. However, FBe (100, 200, and 300 mg kg^− 1^) treated rats showed significant (*p* < 0.01) and dose-dependent increases in SOD activities. Furthermore, the decrease in IND-induced gastric SOD activity was significantly suppressed after a single oral application of FBe (200 mg kg^− 1^) or OM (10 mg kg^− 1^; Table 2).

### Changes in the gastric mucosal histopathology

**Fig. 5:**
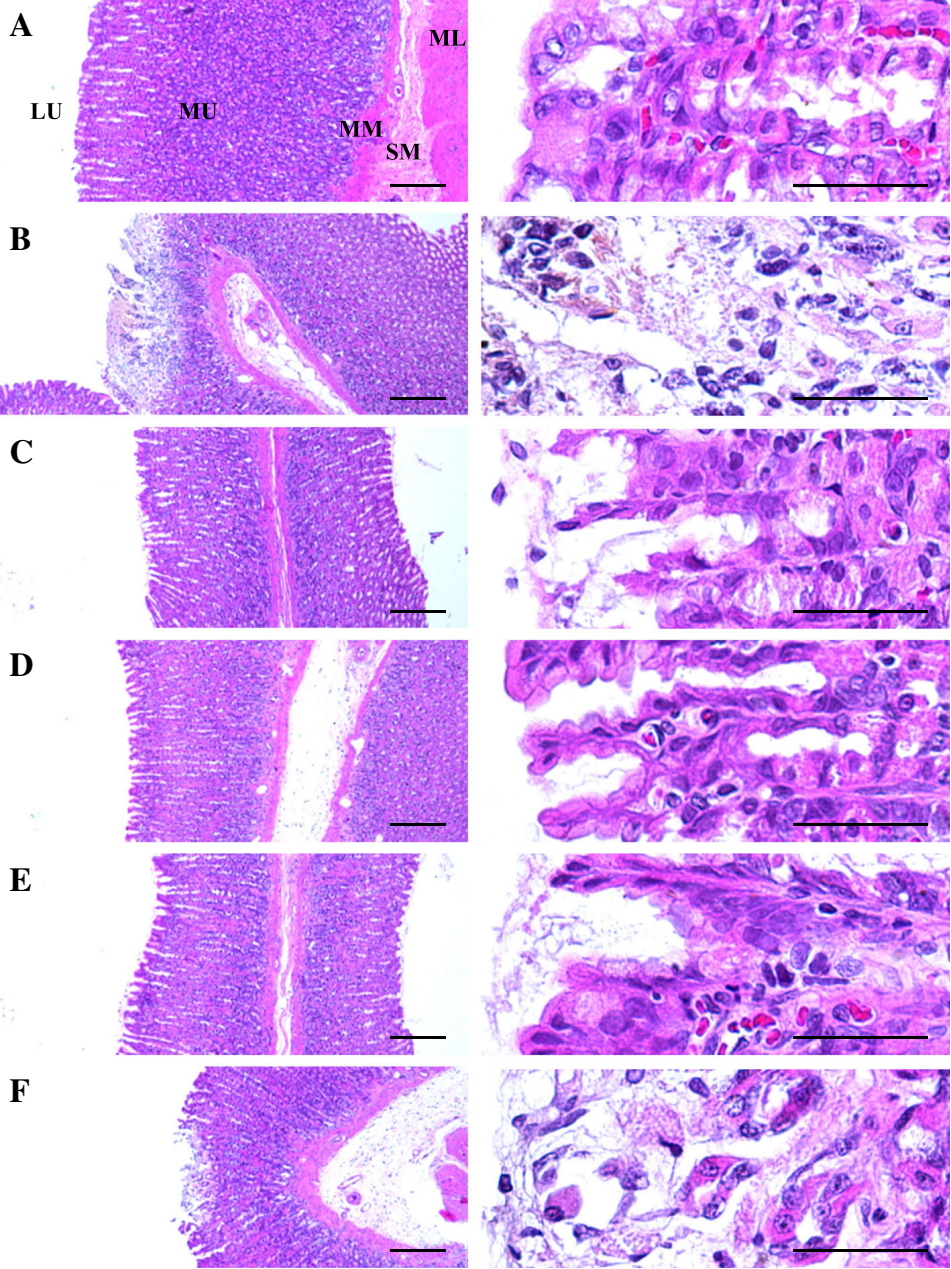
Representative histological images of the fundic mucosa, taken from intact or IND-treated rats. **a**: Intact control (DW 5 mg kg^− 1^, twice administrated rats with 30 min-intervals). **b**: IND control (DW 5 mg kg^− 1^ and IND 25 mg kg^− 1^ administered gastric mucosal damage induced vehicle control rats). **c**: OM (OM 10 mg kg^− 1^ and IND 25 mg kg^− 1^ administered reference drug treated rats). **d**: FBe 300 (FBe 300 mg kg^− 1^ and IND 25 mg kg^− 1^ administered the highest dosage experimental rats). **e**: FBe 200 (FBe 200 mg kg^− 1^ and IND 25 mg kg^− 1^ administered the middle dosage experimental rats). **f**: FBe 100 (FBe 100 mg kg^− 1^ and IND 25 mg kg^− 1^ administered the lowest dosage experimental rats). FBe: triple fermented barley extract as test material; DW: Distilled water; IND: indomethacin; OM: omeprazole; LU: lumen; ML: muscle layer; MM: muscularis mucosa; MU: mucosa layer; SM: submucosa. All hematoxylin and eosin stain. Scale bars = 180 μm

Severe desquamation of focal epithelium, focal extensive superficial epithelial damage, congestion/ hemorrhages, and necrosis of ulcerative lesions and gastric glands were observed following treatment with IND in rats. However, these microscopic ulcerative lesions were significantly suppressed by treatment with test materials. During histomorphometric and semiquantitative analyses, a significant increase in semiquantitative histological scores and percentage of invaded lesions, and a decrease in peri-ulcerative mucosa thickness were noticed in IND control rats compared to the intact vehicle control rats. But, these changes were significantly regulated by treatment with FBe (100, 200, and 300 mg kg^− 1^). Furthermore, a significant decrease in semiquantitative histological scores and percentage of invaded lesions, and an increase in peri-ulcerative mucosa thickness were noticed in OM (10 mg kg^− 1^) administered rats compared to the IND control rats. FBe (200 mg kg^− 1^) revealed inhibitory effect on the IND-induced histopathological gastric mucosal damages which was similar to those noticed in rats treated with OM (10 mg kg^− 1^; Table 3, Fig. 5).

**Table 3:** Histomorphometrical analysis on the fundus, taken from intact or IND-treated rats

Items (Unit) groups	Histomorphometry (at sacrifice)		
	Invasive percentages of lesions* (%)	Mean peri-ulcerative mucosa thickness (μm)	Semiquantitative scores (Max = 3)
Control			
Intact	4.27 ± 2.87	902.66 ± 131.22	0.38 ± 0.52
IND	68.53 ± 12.68^e^	281.50 ± 87.64^a^	2.75 ± 0.46^a^
Reference			
OM 10 mg kg^− 1^	26.65 ± 15.66^e,f^	538.88 ± 87.26^a,b^	1.38 ± 0.52^a,b^
FBe treated as			
100 mg kg^− 1^	32.59 ± 14.44^e,f^	410.09 ± 58.15^a,c^	2.00 ± 0.53^a,b^
200 mg kg^− 1^	25.63 ± 10.67^e,f^	521.04 ± 90.25^a,b^	1.50 ± 0.53^a,b^
300 mg kg^− 1^	13.00 ± 5.46^e,f^	635.59 ± 129.75^a,b^	1.13 ± 0.64^a,b^

Values are expressed as mean ± S.D. of eight rat samples

*FBe* triple fermented barley extract as test material, *IND* indomethacin, *OM* omeprazole

*$$ \mathrm{Invasive}\ \mathrm{percentage}\ \mathrm{of}\ \mathrm{lesions}\ \left(\%\right)=\left(\frac{\mathrm{Length}\ \mathrm{of}\ \mathrm{lesions}\ \mathrm{on}\ \mathrm{the}\ \mathrm{crossly}\ \mathrm{trimmed}\ \mathrm{fundic}\ \mathrm{walls}}{\mathrm{Total}\ \mathrm{thickness}\ \mathrm{of}\ \mathrm{crossly}\ \mathrm{trimmed}\ \mathrm{fundic}\ \mathrm{walls}}\right)\ \mathrm{x}\ 100 $$

Semiquantitative scoring was divided into four groups; 0: normal intact mucosa, 1: slight surface erosive damages, 2: moderate mucosa damages, and 3: severe total mucosa damages

^a^*p* < 0.01 as compared with intact control by LSD test

^b^*p* < 0.01 and ^c^*p* < 0.05 as compared with IND control by LSD test

^d^*p* < 0.01 as compared with intact control by MW test

^e^*p* < 0.01 as compared with IND control by MW test

## Discussion

Mucosal damages can be easily generated by exogenous and endogenous ROS and free radicals [38]. Changes in the gastric mucosal antioxidant defense system have been linked with the pathogenesis and progression of NSAIDs associated gastric ulcers [11]. Indomethacin, an NSAID, is widely approved in medical practice [7]; however, an ulcerative gastrointestinal effect may arise from the inhibition of prostaglandin synthesis [8] and harmful ROS generations [38]. The antioxidants are advocated to offer effective protection against induction and progression of gastric ulcers [39, 40], and the fermentation processes are linked with increased bioavailability of various phenolic compounds and antioxidants [15, 41, 16–18]. The reported medicinal attributes and antioxidant properties of the natural substances prompted our assessment of the possible protective effects of FBe against IND-induced gastric lesions in Sprague-Dawley rat model.

The decrease in the gross hemorrhagic lesion area was considered as an indicator of the protective effects of the test substances on the gastric mucosa corroborating previous efficacy studies [28, 42]. Lesser gross lesions are equal to more favorable protective effects [10]. FBe (200 mg kg^− 1^) inhibited the gastric gross lesion areas and showed similar gastroprotective effects as those observed with OM (10 mg kg^− 1^) in IND-induced gastric mucosal damaged rats. This suggests that FBe dosage can be effectively regulated for patients undergoing various gastric disorders, starting with a dose of 100 mg kg^− 1^.

IND markedly enhanced the MPO activity in stomach tissue whereas all three doses of FBe significantly lowered the MPO activity. FBe (200 mg kg^− 1^) displayed inhibitory effect on the increase of IND-induced MPO activities similar to those observed on treatments with OM. An increase in MPO activity has been reported in NSAID-damaged stomach tissue [40], which shows increased neutrophil secretion in stomach lesions [43]. Excessive secretions of free radicals such as OH, H_2_O_2_, and O_2_ due to the neutrophil secretion cause tissue damages [43]. Therefore, the dose-dependent decreases in MPO activity after a single oral administration of FBe demonstrate that FBe has favorable anti-inflammatory effects, and can suppress the harmful effects of infiltrated neutrophils in gastric mucosa induced by IND treatment.

All tested doses of FBe markedly lowered the lipid peroxidation (MDA content) compared to the IND control. FBe (200 mg kg^− 1^) also decreased the MDA level in a similar manner as OM. The increase in MDA level is associated with increased tissue damage and it is an important cause of gastric damages associated with NSAIDs [44, 45].

GSH and other antioxidant mechanisms regulate the ROS at a specific required cellular concentration which prevents tissue damage [46]. The GSH levels in the stomach tissue of FBe (200 mg kg^− 1^) -administered rats were statistically higher compared to those observed in the IND control rats. Especially, higher GSH levels were observed in the stomach tissues with less damage. This increase in GSH levels is associated with gastroprotective effects of FBe and illustrates that the FBe dosage can be easily regulated for patients suffering from different levels of gastric damages.

Statistically different CAT activity was observed in stomach tissue of the healthy rats and the IND control rats. CAT activity in the FBe (200 mg kg^− 1^) -administered rats decreased significantly in a dose-dependent manner. Previous studies have shown an increasing trend in CAT activity in the IND-induced stomach damages [47, 48]. The increased CAT activity in IND-administered groups shows an increase in H_2_O_2_ while the decreased CAT activity in FBe-administered groups indicates a decreased oxidative stress.

The outcomes of this study support earlier studies stating that NSAIDs lower SOD activity in rat stomach tissues [47, 48]. SOD activity was inhibited in IND-administered rats; however, FBe and OM showed higher SOD activity. FBe (200 mg kg^− 1^) showed an inhibitory effect against IND-induced SOD activity inhibition similar to those observed on treatment with OM. SOD and other antioxidants are important in reducing IND-induced gastric damage as it partially prevents oxidative damages [49]. The relationship between prostaglandin synthesis and SOD activity has been studied in details and it is considered as a possible mechanism of IND-induced ulcers [46, 49].

Gastric mucosal histopathological analysis revealed enhanced focal extensive desquamation of focal epithelium superficial epithelial damage, focal hemorrhages/congestions, neutrophil necrosis and infiltrations of gastric glands, and ulcerative lesions. The findings of this study were similar to the previously reported studies of Bhattacharya et al. [6], and Graziani et al. [50]. Histopathological analysis has been used as a valuable criterion to estimate the gastroprotective effects of new medicinal products, including herbal extracts [10, 51, 52]. In the present study, OM and FBe significantly inhibited the IND associated microscopic ulcerative lesions in a dose-dependent manner. FBe (200 mg kg^− 1^) exhibited a histopathological effect similar to that observed in treatment with OM. The changes were re-confirmed by histomorphometric analysis. Peri-ulcerative mucosal thickness was significantly decreased while the percentage of invaded lesions and the semiquantitative histological scores were prominently increased in IND treatment group. However, the changes were favorably normalized in a dose-dependent manner with FBe and after a single oral application of OM. The changes observed in OM treatment were similar to those observed in treatment with FBe (200 mg kg^− 1^).

The acute toxicity profile of the test material (FBe), analyzed in a previous study by our group [21], showed non-toxic behavior of FBe to mice, and therefore, it was considered likely to be safe for medical use. A single oral application of FBe showed an LD_50_ of > 2000 mg kg^− 1^, which is the KFDA (Korean Food and Drug Administration) recommended dose for both male and female rodents. FBe in this study showed a significant and dose-dependent decrease in IND-induced gastric damage, the hemorrhagic gross lesion, gastric MPO content, and histopathological gastric ulcerative lesion. Additionally, FBe dose-dependently strengthened the antioxidant defense systems, lowered lipid peroxidation level, and CAT activity but enhanced the SOD activity and GSH content. Similar results were obtained in our previous study on the gastroprotective effect of FBe on HCl/Et-OH-induced gastric mucosal damage in mice [53]. FBe (200 mg kg^− 1^) showed promising gastroprotective effects in the present study on indomethacin-induced gastric ulcer in rats and in the previous study on HCl/Et-OH-induced gastric ulcer in mice [53]. The inhibitory effects were similar to the commercially available anti-ulcer drugs, such as ranitidine (100 mg kg^− 1^) and omeprazole (10 mg kg^− 1^). In our previously study, FBe (200 and 300 mg kg^− 1^) also indicated satisfactory laxative effects, mediated by the increase in gastrointestinal motility in normal rats [20]. Hence, FBe, as a potent food supplement, can be suitable for patients suffering from gastric mucosal disorders.

The findings of this study are considered as direct evidence that oral administration of FBe had promising gastroprotective effects by strengthening the anti-inflammatory effects and the innate antioxidant defense system. It has been reported that the polyphenols and total flavonoids have potent antioxidant and anti-inflammatory effects. Thus, the total polyphenols and flavonoids contents of FBe may be responsible for the anti-inflammatory, antioxidant, and gastroprotective effects of FBe. Further research is needed to elucidate the specific polyphenols and flavonoids responsible for its antioxidant and anti-inflammatory effects.

## Conclusions

By evaluating the important parameters for protective effects on the Sprague-Dawley rats with IND-induced gastric mucosal damage, during this study oral supply of FBe (100, 200, and 300 mg kg^− 1^) revealed promising gastroprotective effects by strengthening the anti-inflammatory effects and the antioxidant defense system. Moreover, the experimental results also showed that FBe (200 mg kg^− 1^) had similar inhibitory effects against IND-induced gastric damage in rats to those observed in treatment with OM (10 mg kg^− 1^), indicating that FBe dosage can be easily regulated for patients suffering from different levels of gastric damages, starting at a dosage of 100 mg kg^− 1^ as observed in IND-induced gastric damages in rats.

## Abbreviations

ATCC: American type culture collection
CAT: catalase
EDTA: ethylene diamine tetraacetic acid
FBe: triple fermented barley extract
GSH: glutathione
H&E: hematoxylin and eosin stain
HETAB: hexadecyltrimethylammonium bromide
IND: indomethacin
KACC: Korean Agricultural Culture Collection
MDA: malondialdehyde
MPO: myeloperoxidase
NBF: neutral buffered formalin
NSAIDs: non-steroidal anti-inflammatory drugs
OM: omeprazole
PPI: proton pump inhibitor
ROS: reactive oxygen species
SOD: superoxide dismutase
